# On-chip wavelength division multiplexing filters using extremely efficient gate-driven silicon microring resonator array

**DOI:** 10.1038/s41598-023-32313-0

**Published:** 2023-03-31

**Authors:** Wei-Che Hsu, Nabila Nujhat, Benjamin Kupp, John F. Conley, Alan X. Wang

**Affiliations:** 1 School of Electrical Engineering and Computer Science, Oregon State University, Corvallis, OR 97331 USA; 2 Department of Electrical and Computer Engineering, Baylor University, One Bear Place #97356, Waco, TX 76798 USA

**Keywords:** Silicon photonics, Integrated optics

## Abstract

Silicon microring resonators (Si-MRRs) play essential roles in on-chip wavelength division multiplexing (WDM) systems due to their ultra-compact size and low energy consumption. However, the resonant wavelength of Si-MRRs is very sensitive to temperature fluctuations and fabrication process variation. Typically, each Si-MRR in the WDM system requires precise wavelength control by free carrier injection using PIN diodes or thermal heaters that consume high power. This work experimentally demonstrates gate-tuning on-chip WDM filters for the first time with large wavelength coverage for the entire channel spacing using a Si-MRR array driven by high mobility titanium-doped indium oxide (ITiO) gates. The integrated Si-MRRs achieve unprecedented wavelength tunability up to 589 pm/V, or V_π_L of 0.050 V cm with a high-quality factor of 5200. The on-chip WDM filters, which consist of four cascaded ITiO-driven Si-MRRs, can be continuously tuned across the 1543–1548 nm wavelength range by gate biases with near-zero power consumption.

## Introduction

Silicon photonics has become the most promising platform to enable high bandwidth and energy-efficient optical interconnects for data centers and high performance computing systems^1^. To meet the grand challenge of bandwidth density, on-chip wavelength division multiplexing (WDM) optical interconnects are proposed and demonstrated for highly parallel optical interconnect systems^2–4^. For on-chip WDM optical interconnects, silicon microring resonators (Si-MRRs) can function both as wavelength filters and as electro-optic modulators with great advantages in ultra-compact footprint and high energy efficiency^2,5,6^. A typical on-chip WDM module can be formed by coupling an array of Si-MRRs with different resonant wavelengths (λ_res_) to a single bus waveguide, and each Si-MRR can be independently tuned and modulated^3^. However, the λ_res_ of a Si-MRR is very sensitive to the variation of fabrication processes and temperature fluctuations^7,8^. It is essential to precisely control the working wavelength of each Si-MRR. In past years, free carrier injection using PIN diodes or thermal heaters have been adopted for extensive λ_res_ tuning^9–15^, but typically require a high power consumption of 3–4 mW/nm for carrier injection and 4 mW/nm for thermal tuning.

To address the grand challenge faced by future large-scale optical interconnect systems, we demonstrate in this article the first gate-tuning on-chip WDM filters showing a large wavelength coverage of the entire channel spacing among all Si-MRR filters. Compared with previously reported on-chip WDM modules, gate-driven Si-MRRs can achieve wavelength tuning with near-zero power consumption^16^. The on-chip WDM filters herein consist of four cascaded tunable Si-MRRs with metal–oxide–semiconductor (MOS) gates formed by a high mobility transparent conductive oxide (TCO), which show much larger electro-optic (E-O) efficiencies than reversed PN junctions. It can realize a large wavelength tuning range with a low gate voltage and negligible power consumption. Moreover, MOS-driven silicon photonic devices can be heterogeneously integrated with other semiconductor materials to achieve even better E-O efficiency, such as III–V compound semiconductors and TCOs^17,18^. The tunable Si-MRRs integrated with III-Vs and TCOs have experimentally demonstrated significantly higher E-O wavelength tunability with thin high-κ hafnium oxide (HfO_2_) insulators^19,20^.

Our group chose a high mobility TCO material as the gate for the tunable Si-MRR array. TCO materials can be conformally deposited on the microring waveguide to cover the top and sidewalls of the waveguide, which provides a better overlap between the optical mode in the waveguide and accumulated carriers to enhance the E-O efficiency compared to III–V compound semiconductors that are bonded on top of the waveguide^16^. Previously, we demonstrated a tunable Si-MRR gated by indium-tin-oxide (ITO), which achieves an E-O wavelength tunability of 271 pm/V using a 16 nm-HfO_2_ insulator^20^, a photonic crystal nanocavity, and electro-absorption modulators^21,22^. However, the quality factor (Q-factor) of ITO-gated Si-MRR was limited to 1000 due to the low carrier mobility, which caused a high optical absorption. Such a low Q-factor restricts the channel density of the WDM module^23^. In principle, high mobility TCO gates can reduce the optical absorption and enhance the Q-factor of tunable Si-MRRs^24^. We have also experimentally reported a tunable Si-MRR with high mobility titanium-doped indium oxide (ITiO) to achieve a high Q-factor of 12,000^25^. This device also allowed electrical tuning of the λ_res_ to compensate for temperature variations with low power consumption. However, only a moderate E-O wavelength tunability of 130 pm/V was achieved since the microring had a wide waveguide width of 400 nm, and the optical mode did not have enough overlap to interact with the accumulated carriers.

To achieve the best performance, in this work, we systematically investigated how the waveguide width and the mobility of the TCO gate would impact the Si-MRR, especially in terms of E-O wavelength tunability and Q-factor. We conclude that the tunable Si-MRR driven by high mobility ITiO gate with a reduced microring waveguide width of 300 nm is preferred to achieve both high E-O wavelength tunability and high Q-factors, which is suitable for on-chip WDM filters. Hence, four tunable Si-MRRs with such a structure are cascaded to form on-chip WDM filters in the experiment. The individual ITiO-driven Si-MRR can achieve extremely high E-O wavelength tunability up to 589 pm/V with a high Q-factor of 5200. In addition, the optical field-effect mobility (μ_op,FE_) in the accumulation layer of ITiO is experimentally measured, rising from 40 to 70 cm^2^/V/s with increasing gate biases. The increased mobility benefits the tunable Si-MRR to maintain the high Q-factor. Moreover, with the large wavelength tunability, such on-chip WDM filters can be continuously tuned across the 1543–1548 nm wavelength range by gate biases with near-zero power consumption. They can maintain uniform channel spacing, which can be influenced by fabrication errors.

## Results

### Device design

**Figure 1:**
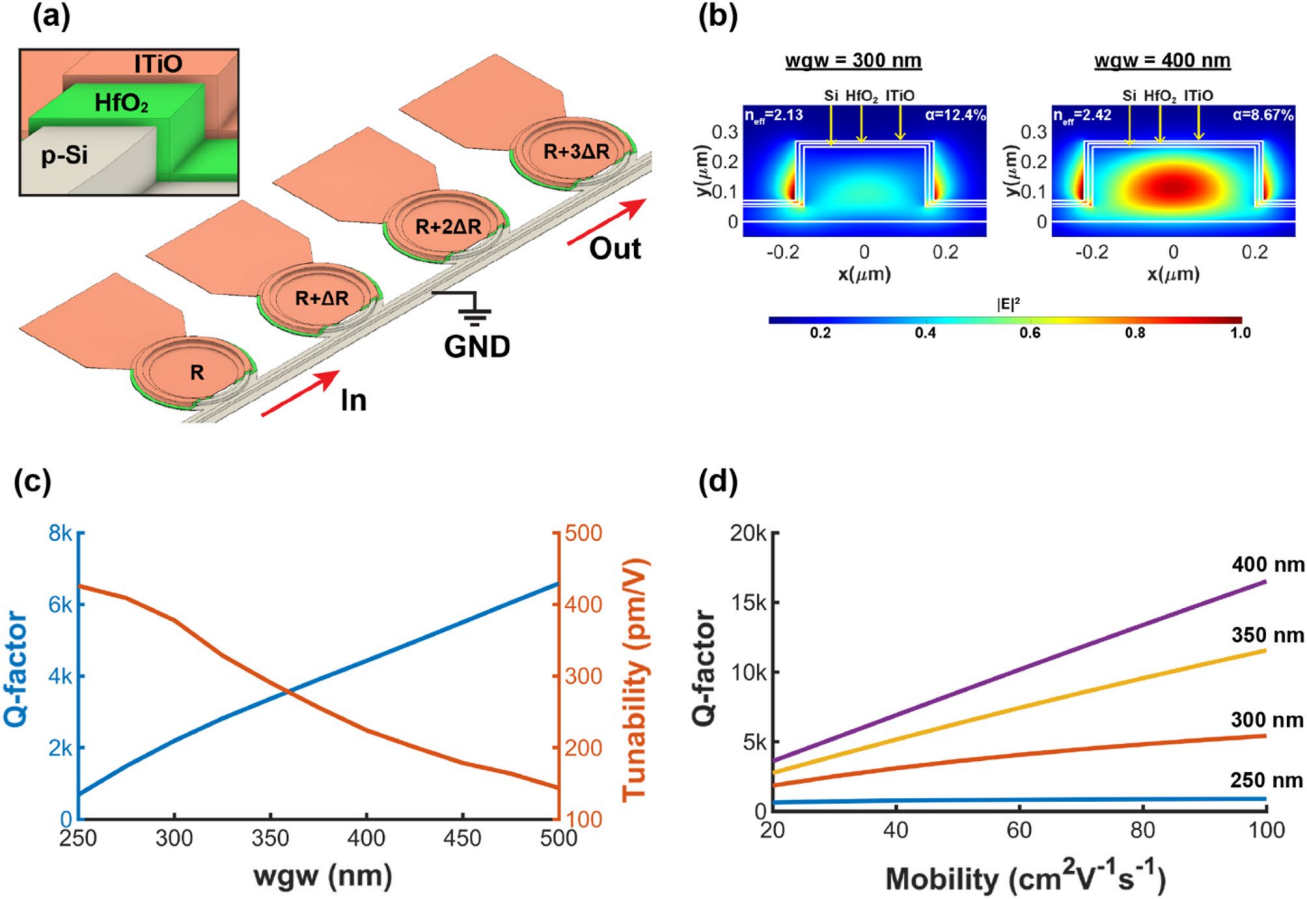
(**a**) 3D schematic of the on-chip WDM filters consisting of four cascaded tunable Si-MRRs with different radii. Inset: zoom-in view of microring waveguide with ITiO/HfO_2_/Si MOS capacitor. (**b**) Simulated mode profiles in the active region with waveguide widths of 300 and 400 nm, respectively. (**c**) Simulated Q-factor (blue) and tunability (orange) versus waveguide widths by assuming that the gate material has a carrier concentration of 3 × 10^19^ cm^−3^ and mobility of 25 cm^2^V^−1^ s^−1^ in the simulation. (**d**) Simulated Q-factor as a function of mobility with different waveguide widths, assuming that the bent Si waveguide has a radius of 8 μm and a height of 250 nm with a 50 nm slab in the simulation.

The on-chip WDM filters consist of four cascaded tunable Si-MRRs with different radii, as illustrated in Fig. 1a. The microring waveguides of the tunable Si-MRRs are formed by the ITiO/HfO_2_/Si MOS capacitors, as shown in the inset of Fig. 1a. When the negative bias is applied to the ITiO gate, the optical mode interacts with the accumulated carriers, and the λ_res_ of Si-MRR has a blue shift^20,25^. Figure 1b shows the simulated mode profiles in the cross-section of the tunable Si-MRR waveguides. The overlapping factor, which describes the overlap between the optical mode and accumulation layer, is defined as^26^:

1 $$\alpha = \frac{\int\Delta {N}_{c}q\varepsilon {\left|E\right|}^{2}dA}{{\mathrm{Q}}_{\mathrm{tot}}\mathrm{max}({\varepsilon \left|E\right|}^{2})},$$

where $\varepsilon$ is the permittivity distribution, and E is the electric field distribution of the optical mode. $\Delta {N}_{c}$ is the change of free carrier concentration, and ${\mathrm{Q}}_{\mathrm{tot}}$ is the total change of the charge.

Figure 1b also shows the calculated overlapping factor for the waveguide widths of 300 and 400 nm, respectively. The narrower waveguide provides a better overlapping factor and induces a larger index modulation ($\Delta {n}_{eff}$) to improve the E-O wavelength tunability. The E-O wavelength tunability can be expressed as^20^:

2 $$Tunability= -\frac{{\lambda }_{res}}{{n}_{eff}}\frac{\Delta {n}_{eff}}{\Delta V},$$

where n_eff_ is the effective index of the waveguide, and $\frac{\Delta {n}_{eff}}{\Delta V}$ is the change of the n_eff_ caused by the gate voltage change.

Since the narrower waveguide has a lower n_eff_, it further increases the E-O wavelength tunability. Therefore, the narrower waveguide enhances the E-O wavelength tunability but concomitantly reduces the Q-factor due to the increased optical absorption from the accumulated carriers, as shown in Fig. 1c. To overcome this problem, the simulation in Fig. 1d shows that high mobility TCOs can reduce the optical absorption loss to improve the Q-factor. However, for the extremely narrow waveguide such as 250 nm, the Q-factor is majorly limited by the bending loss and is not significantly improved by the high mobility gate material. Therefore, the 300 nm waveguide width heterogeneously integrated with the high mobility TCO, such as ITiO^27^, is preferred to achieve both a high E-O wavelength tunability and a high Q-factor.

### Characterization of an individual tunable Si-MRR

**Figure 2:**
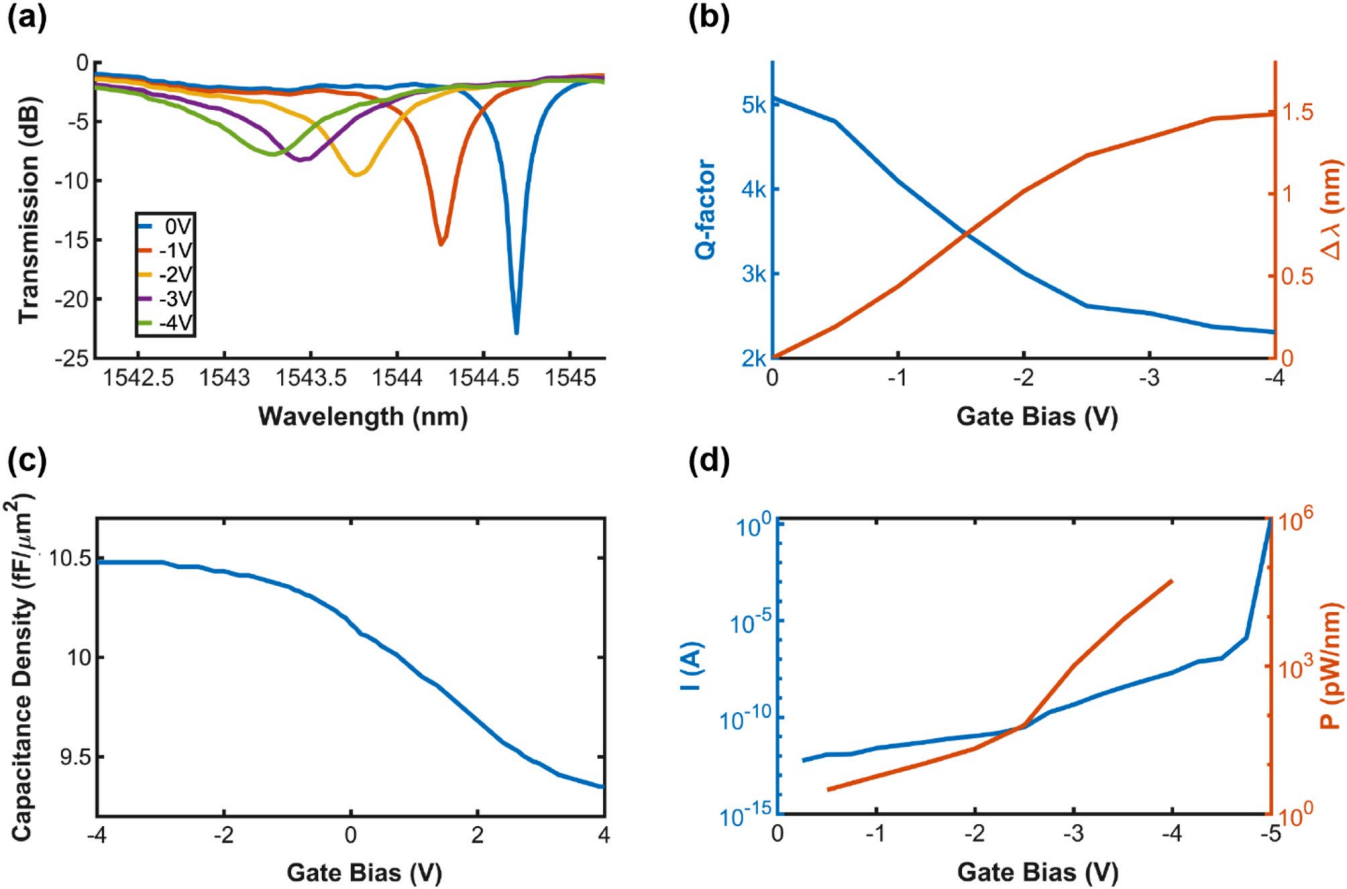
(**a**) Normalized transmission spectra of a tunable Si-MRR with different applied gate biases. (**b**) Measured Q-factor (blue) and Δλ (orange) as a function of the gate bias. (**c**) Measured capacitance density of a metal/9 nm-HfO_2_/Si MOS capacitor as a function of gate bias. (**d**) The leakage current of a tunable Si-MRR (blue) and calculated power consumption (orange).

Figure 2a shows the normalized transmission spectra of an individual tunable Si-MRR with different applied biases. The individual tunable Si-MRR is designed under the critical coupling condition at 0 V. When a negative bias applies to the ITiO gate, it blue-shifts the λ_res_ and increases the optical absorption in the microring waveguide, which reduces the Q-factor. Besides, the increase of the optical absorption influences the coupling condition of the Si-MRR, so the extinction ratio (ER) varies with the applied bias. Figure 2b shows the measured Q-factor and λ_res_ shift (Δλ) at different applied biases. The individual tunable Si-MRR has a high Q-factor of 5200 at 0 V, and the Q-factor gradually decreases with the Δλ caused by the negative bias. It has an electrical wavelength tuning range of 1.5 nm from 0 V to – 4 V, and the maximum E-O wavelength tunability of 589 pm/V occurs at – 1 V, corresponding to an ultra-small V_π_L of 0.050 V cm. Figure 2c shows the capacitance density as a function of gate bias for metal/9 nm-HfO_2_/Si MOS capacitor. It has a capacitance density of 10.48 fF/μm^2^ at – 4 V, and the overall capacitance of the individual tunable Si-MRR can be calculated to be 540 fF at – 4 V. Figure 2d shows the leakage current of the individual tunable Si-MRR, and the power consumption is calculated with respect to the E-O wavelength tunability. It has a near-zero electrical power consumption from pW/nm to nW/nm, which is at least 10^6^× more power-efficient than the free carrier injection or thermal tuning (mW/nm).

**Figure 3:**
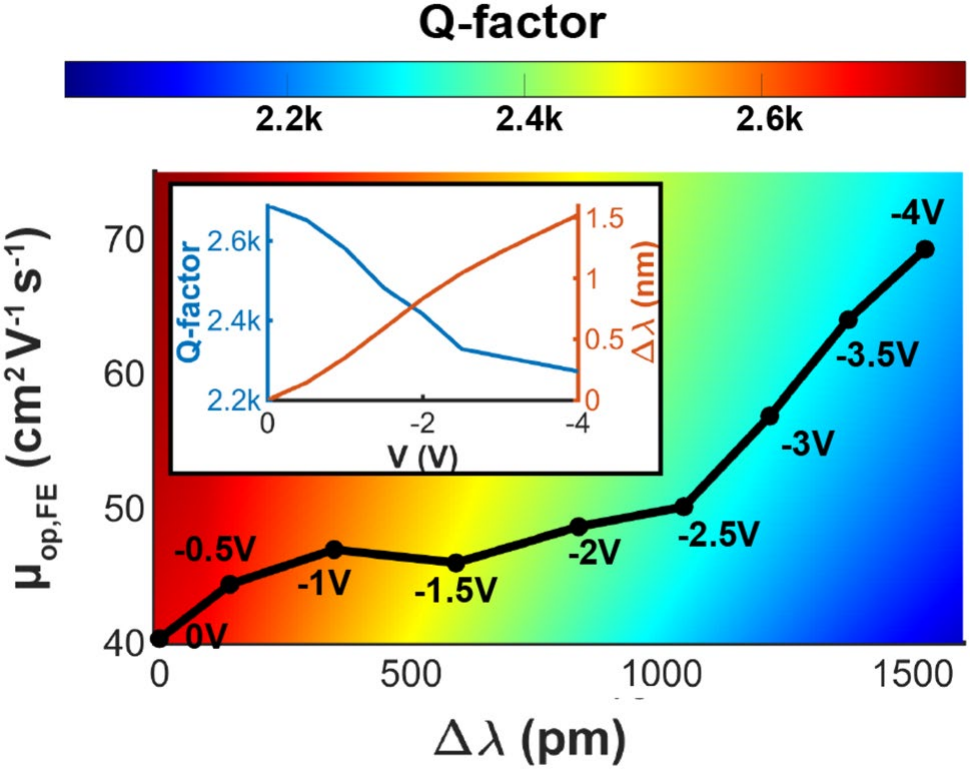
Extracted μ_op,FE_ from the experimental Q-factor and Δλ. ITiO has an initial carrier concentration of 3.3 × 10^19^ cm^−3^ and μ_bulk_ of 44 cm^2^/V/s from Hall measurement. The inset figure shows the experimentally measured Q-factor and Δλ that are similar to Fig. 2b.

Meanwhile, when the negative bias increases, the μ_op,FE_ in the accumulation layer of ITiO rises. This has a great benefit in reducing optical absorption loss and maintaining the Q-factor^28^. To characterize the μ_op,FE_, the ITiO is deposited at the same RF sputtering conditions on another individual tunable Si-MRR, and the ITiO has the bulk mobility (μ_bulk_) of 44 cm^2^/V/s from the Hall effect measurement. Figure 3 shows the experimental μ_op,FE_ of the ITiO, obtained from the measured Q-factor and Δλ (in the inset of Fig. 3). The initial μ_op,FE_, at 0 V is 40 cm^2^/V/s, slightly lower than the μ_bulk_. When the negative bias rises to − 4 V, μ_op,FE_ reaches 70 cm^2^/V/s.

### Characterization of the on-chip WDM filters

**Figure 4:**
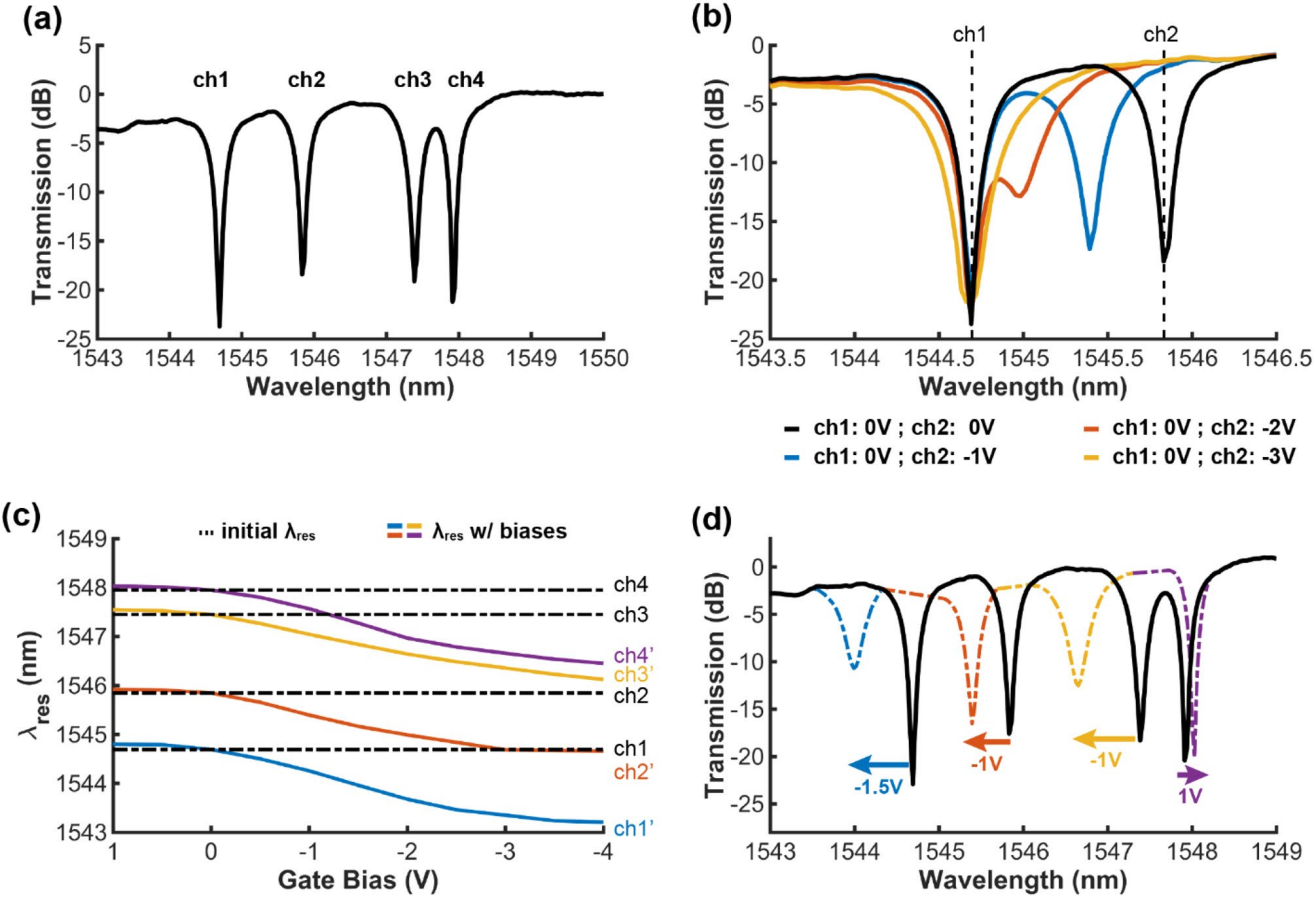
(**a**) Normalized transmission spectra of the on-chip WDM filters. The λ_res_ of four rings are marked. (**b**) Zoom in transmission spectra for ch1 and ch2 and electrical tuning of ch2 with gate biases. The black curve is the spectrum at 0 V, and color curves show the different biases applied on ch2. (**c**) λ_res_ map with the applied biases. The dashed lines show the initial λ_res_ of channels (at 0 V), and the color curves show the λ_res_ of channels shift. (**d**) Transmission spectrum of the on-chip WDM filters showing the application of gate bias on each channel (color dashed curves) to compensate for the non-uniform channel spacing caused by fabrication errors (black solid curve).

The on-chip WDM filters consist of four cascaded tunable Si-MRRs with slightly different radii, so each tunable Si-MRR has a different λ_res_ position, and the four resonant dips (four channels) can be fitted into the free spectral range (FSR) of 11 nm, as shown in Fig. 4a. Although the off-resonant state of the on-chip WDM filters is not at 0 dB, which is due to imperfect normalization to the reference waveguide (without resonators), the result does not affect the characterization of wavelength tuning. Besides, it is noteworthy that the fabricated on-chip WDM filters do not have uniform spacing between each channel due to fabrication quality since the λ_res_ of Si-MRR is very sensitive to process variations. Each individual tunable Si-MRR can achieve high E-O wavelength tunability and a large wavelength tuning range, so the position of the λ_res_ can be electrically tuned from one channel to the other channel. For example, as shown in Fig. 4b, the resonant dip of ch2 can be tuned to ch1 with an applied bias of − 3 V. Figure 4c shows the λ_res_ tuning map with applied biases on each gate. The dashed lines show the initial λ_res_ of each channel at 0 V. Each channel of the on-chip WDM filters can be electrically tuned with moderate gate biases. Overall, the fabricated on-chip WDM filters can be consecutively tuned across the 1543–1548 nm wavelength range by gate biases, as presented in the solid curves. As illustrated in Fig. 4d, applying gate biases can compensate for the non-uniform channel spacing of the on-chip WDM filters caused by fabrication errors. The solid black curve shows the initial spectrum of the fabricated on-chip WDM filters, which is without any applied biases (0 V). The dashed color curves show that each channel can be independently tuned by the gate bias, and different colors represent the shift of each channel. Finally, the on-chip WDM filters can be fine-tuned to achieve uniform channel spacing with applied gate biases.

## Discussion

This work experimentally demonstrated the first on-chip gate-driven WDM filters using a Si-MRR array with ITiO/HfO_2_/Si MOS capacitors, showing a large wavelength coverage of the entire channel spacing. Through optimization of device design using high mobility ITiO gate and 300 nm narrow waveguide, the individual tunable Si-MRR could achieve an extremely high wavelength tunability of 589 pm/V (0.050 V cm) with a high Q-factor of 5200. In addition, the μ_op,FE_ of ITiO in the accumulation layer rose with increasing negative gate bias, achieving 70 cm^2^/V/s carrier mobility, which benefits the tunable Si-MRRs to maintain the Q-factor. Each channel in the fabricated on-chip WDM filters could be independently tuned by applying gate bias, achieving continuous wavelength coverage from 1543 to 1548 nm with near-zero power consumption. We also proved that gate biases could compensate for fabrication errors to maintain uniform channel spacing of the four cascaded Si-MRRs. We expect that the demonstrated on-chip WDM filters will serve as a key functional unit for future optical interconnects due to their ultra-high energy efficiency.

## Methods

The on-chip WDM filters are fabricated on a silicon-on-insulator (SOI) wafer using electron beam lithography and reactive ion etching. The Si-MRR array has radii of 8.00 μm, 8.03 μm, 8.06 μm, 8.09 μm, respectively. Each Si-MRR has a narrow waveguide width of 300 nm and a waveguide height of 250 nm with a 30 nm slab. The 10 nm HfO_2_ insulator layer is chosen for our design since it provides a high capacitance density to improve the E-O efficiency and secures the acceptable low leakage current^29^. The HfO_2_ is formed on the entire SOI substrate by atomic layer deposition (ALD) in a Picosun R150 reactor using Tetrakis (ethylmethylamino) hafnium (TEMA-Hf) as the precursor and H_2_O as the oxygen source, and the 9 nm HfO_2_ is on the SOI substrate measured by an ellipsometer (Film Sense FS-1). A 10 nm ITiO layer is deposited by RF sputtering at room temperature as the top gate of the MOS capacitors. Before the metal deposition, the HfO_2_ layer in the Si contact region is etched by a buffered oxide etchant (BOE). Finally, Ni/Au electric pads are thermally evaporated to form contacts with the ITiO gates and the Si bottom substrate of the MOS capacitors.

**Figure 5:**
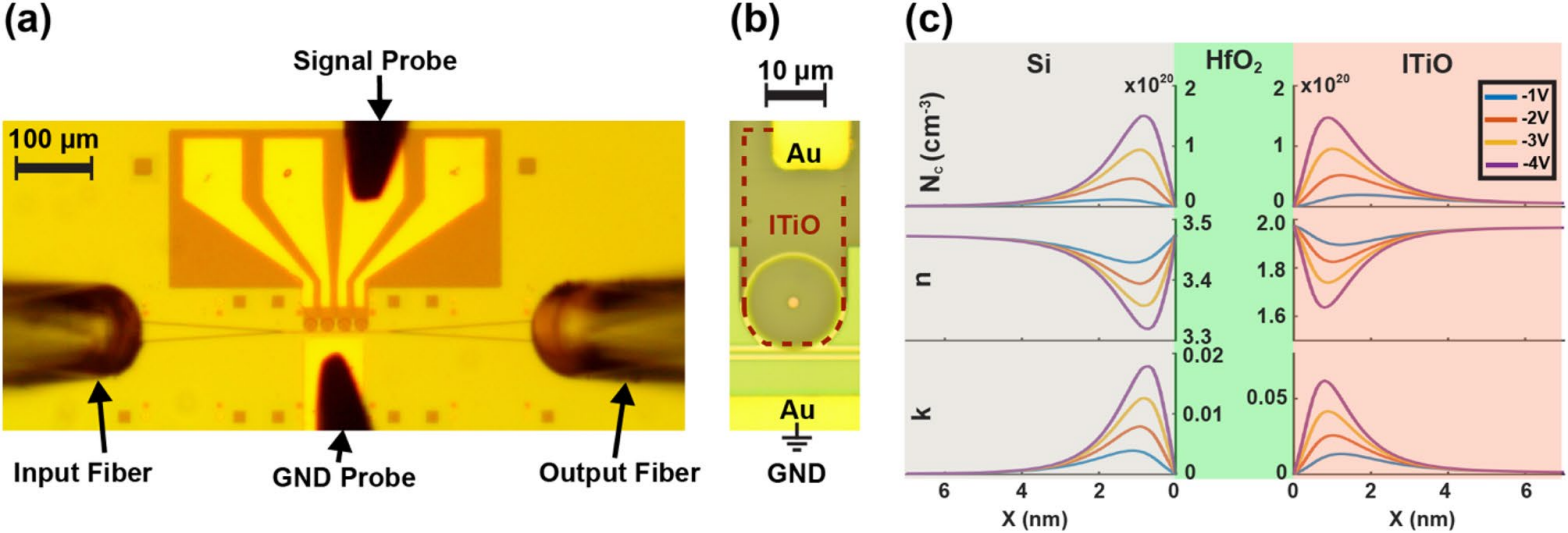
(**a**) Optical microscope image of the fabricated on-chip WDM filters consisting of four cascaded tunable Si-MRRs and testing setup (**b**) Zoom-in view of the individual tunable Si-MRR of the on-chip WDM filters. The dashed line highlights the ITiO gate. (**c**) The simulated carrier concentration (N_c_), refractive index (n), and extinction coefficient (k) distributions with different applied biases at the ITiO/HfO_2_ and the Si/HfO_2_ interfaces.

The fabricated on-chip WDM filters and testing setup are shown in Fig. 5a. The input and output fibers have a tilt angle of 10°, and the light is coupled into and out from the on-chip WDM filters through the grating couplers. The GND probe serves as the common ground for the on-chip WDM filters, and each channel is independently tuned using the gate bias through the single probe. Figure 5b shows the zoom-in view of the on-chip WDM filters, and the ITiO gate covers the active region of each Si-MRR. When the negative bias is applied, the accumulation of free carriers induces the change of refractive index and optical absorption originating from ITiO/HfO_2_ and Si/HfO_2_ interfaces, as shown in Fig. 5c.

## Supplementary Information

Supplementary Information.

## Data Availability

All data generated or analyzed during this study are included in this published article and its supplementary information files.
